# Continuous passive movement does not influence motor maps in healthy adults

**DOI:** 10.3389/fnhum.2015.00230

**Published:** 2015-04-29

**Authors:** Michelle N. McDonnell, Susan L. Hillier, George M. Opie, Matthew Nowosilskyj, Miranda Haberfield, Gabrielle Todd

**Affiliations:** ^1^International Centre for Allied Health Evidence, Sansom Institute for Health Research, School of Health Sciences, University of South AustraliaAdelaide, Australia; ^2^Department of Physiology, The University of AdelaideAdelaide, Australia; ^3^School of Pharmacy and Medical Sciences and Sansom Institute for Health Research, University of South AustraliaAdelaide, Australia

**Keywords:** motor mapping, TMS, passive movement, corticomotor representation, weakness

## Abstract

Hand weakness following stroke is often associated with a reduced representation of the hand in the primary motor cortex. Meaningful sensory input can induce sensorimotor reorganization in the brain, but the after-effect of continuous passive motion (CPM) on the cortical representation is unknown. The purpose of this study was to determine whether repeated sessions of continuous passive movement of the thumb induce a lasting increase in the motor cortical representation of a thumb muscle in healthy adults. Thirteen right-handed healthy adults (mean age 24.3 ± 4.3 years) participated in the study. Single-pulse Transcranial Magnetic Stimulation (TMS) was delivered over the motor area of the target muscle (abductor pollicis brevis) before and/or after a thirty minute session of thumb CPM administered on three consecutive days. TMS was also delivered 5 days after cessation of the CPM intervention. The response to TMS (motor evoked potential) was recorded in the target muscle with surface EMG. Resting motor threshold (RMT), motor evoked potential amplitude at a specified intensity, and the area and volume of the cortical representation of the target muscle were measured. Thumb CPM had no significant effect on TMS parameters (*p* > 0.05 all measures) and performance of an attention task remained unchanged within and across CPM sessions. The results suggest that three sessions of repetitive passive thumb movement is not sufficient to induce a change in the cortical representation of the thumb and is unlikely to reverse the decreased representation of the affected hand following stroke.

## Introduction

Stroke remains the leading cause of adult disability worldwide. Recovery following stroke is variable but only a third of patients regain some dexterity in the first 6 months (Kwakkel et al., 2003) and the majority of stroke survivors are left with residual motor deficits (Duncan et al., 1992). Upper limb deficits limit the ability to perform activities of daily living, primarily due to muscle weakness. This loss of independence contributes to increased healthcare costs and burden of stroke on the individual and the community (Deloitte Access Economics, 2013).

Transcranial magnetic stimulation (TMS) has been used to investigate properties of the corticomotoneuronal projection and the potential for upper limb recovery post-stroke. When TMS is performed in the early stages post-stroke, the size of the evoked response in the weak hand is predictive of recovery of finger control (Turton et al., 1996). However, most patients have small responses to TMS over the affected motor cortex, which is indicative of reduced excitability of cortical projections and/or a decreased cortical representation of the affected hand following stroke (Rossini and Dal Forno, 2004).

The representation of muscles within the motor cortex can be investigated with TMS by moving the stimulating coil over successive positions of the scalp and recording the size of the evoked response in the target muscle (Cramer and Bastings, 2000). The resultant map is relatively stable over time in healthy individuals (Uy et al., 2002) but can change as a result of rehabilitation following stroke (Traversa et al., 1997, 1998; Liepert et al., 2000) or specific training interventions (Liepert et al., 1998). The resultant change in the map is thought to represent reorganization of the motor cortex.

Reorganization of motor cortex can occur following repeated, meaningful sensory input in healthy adults. Combined stimulation of sensory receptors in the hand and the hand representation of the motor cortex, repeated daily, results in expansion of the hand motor map (McKay et al., 2002). Interestingly, the motor map expansion persists for at least 2 days post-stimulation (McKay et al., 2002). The current study sought to investigate if continuous passive movement (CPM, used regularly following orthopedic surgery) is also capable of evoking expansion of the motor cortical representation of the muscles involved. Passive movements are associated with acute changes in corticomotoneuronal excitability in healthy adults (Chye et al., 2010), largely due to increased muscle spindle firing rates during muscle lengthening. Furthermore, use of blood oxygen level-dependent magnetic resonance imaging reveals substantial reorganization of sensorimotor cortex in healthy adults following daily passive movement of the wrist (duration 20 min) for 1 month (Carel et al., 2000). A single session of passive movement of the wrist can also alter the corticomotor representation of a forearm muscle, with increased volume of the motor map of the flexor carpi radialis muscle in healthy adults, but not participants following stroke (Lewis and Byblow, 2004). However, the after-effects of repeated passive movement of the thumb on corticomotoneuronal excitability has not been investigated.

The aim of the current study was to determine if three consecutive days of passive thumb movement (30 min per day), increases the representation of thumb muscles in the motor cortex in healthy adults following the intervention. We also investigated if the change in the representation of the thumb muscles was still evident 5 days after cessation of the passive movement paradigm. The thumb was chosen because opposition of the thumb is an essential requirement for functional use of the hand, which is often impaired after stroke. It was hypothesized that repeated afferent input, with attention directed to the thumb, could transiently enlarge the cortical representation of the thumb. If the results of the study support the hypothesis, then use of CPM on the weak hand following stroke could prevent a reduction in the cortical representation of the weak hand muscles and thus aid rehabilitation.

## Materials and Methods

### Participants

Thirteen healthy adults (7 men, 6 women) aged 20–33 years participated in the study. Inclusion criteria were right-hand dominant (according to the Edinburgh Handedness Inventory (Oldfield, 1971)), no neurological or orthopedic conditions affecting the hand, and no contra-indications to TMS (Rossi et al., 2009). All participants provided written, informed consent in accordance with the Declaration of Helsinki and the study was approved by the local institutional ethics committee.

### Testing Procedures

A close-fitting flexible cap was placed on the participants head. The cap was marked with a grid of 1 cm spacing. TMS was delivered over points on the grid using a Magstim 200^2^ stimulator and a figure-of-eight coil (part number: 3281–00, 90 mm external diameter of wings, Magstim, Whitland, UK). The coil was initially placed over the motor cortex in the left hemisphere, over the abductor pollicis brevis (APB) motor area. The handle of the coil pointed posteriorly at approximately 45 degrees to the midline and tangentially to the skull. This coil position induces a posterior-to-anterior current in the brain and is optimal for stimulating the hand region of the motor cortex. Single stimuli were delivered at a rate of ~0.2 Hz.

The response to TMS (motor evoked potential or “MEP”) in the target muscle (right APB) was recorded with electromyography (EMG). Two surface EMG electrodes (Ag-AgCl, 10 mm diameter) were placed over the muscle belly and tendon. EMG signals were sampled at 2000 Hz, amplified (1000×), and filtered (20–1000 Hz) using a data acquisition system (1902 with Power 1401 interface and Signal software, Cambridge Electronic Design, Cambridge, UK).

The experiment began with determination of the optimal scalp site for eliciting a MEP for APB. The optimal site was marked and resting motor threshold (RMT) was determined. Determination of RMT involved initially setting the intensity of stimulation well above threshold and then reducing the intensity in steps of 1–2% of stimulator output until it was below threshold. RMT was defined as the stimulus intensity that produced an MEP of amplitude greater than 0.05 mV in 5 out of 10 consecutive stimuli. Fifteen stimuli were then delivered during complete muscle relaxation at an intensity of 120% RMT. Visual feedback was provided via a computer screen to ensure complete muscle relaxation and any trials with pre-stimulus voluntary EMG activity were discarded from the analysis. After collection of MEPs evoked at a stimulus intensity of 120% RMT, participants underwent mapping of the cortical representation of the right APB muscle during relaxation, similar to the method described by Uy et al. (2002). Five stimuli were delivered at each scalp site at an intensity of 110% RMT. The first scalp site was the optimal site (APB hot spot) and selection of subsequent sites involved moving the coil outwards in 1 cm steps until no MEPs were elicited. The number of scalp sites that exhibited a MEP amplitude ≥ 0.05 mV is an index of the area of the cortical representation of APB. The sum of the averaged MEP amplitude evoked at all active scalp sites is an index of the volume of the cortical representation of APB (Cicinelli et al., 2006; Meesen et al., 2011). The center of gravity for the map was also calculated according to the method described by Wassermann et al. (1992), using the following formula:

$$\text{CoG}=\sum v_{i}x_{i}/\sum v_{i},\sum v_{i}y_{i}/\sum v_{i},\text{ for scalp sites }x_{i},y_{i}\text{ and amplitudes }v_{i}$$

The between-session difference in the CoG was also calculated.

The above TMS procedure was performed at baseline, following each day of CPM (days 1–3), and 5 days after the final session of CPM (day 8). The distance from the vertex to the nasion, inion, and bilateral pre-tragus was recorded during the baseline session to enable consistent cap (and thus coil) placement on subsequent days.

### Continuous Passive Motion

**Figure 1:**
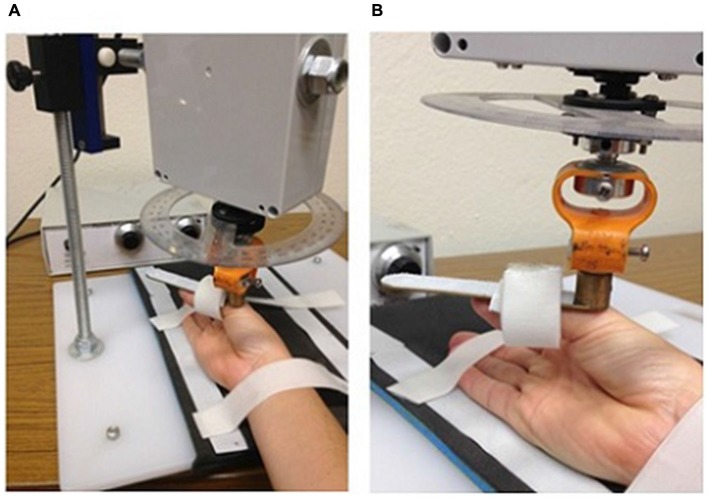
**Custom-made device to passively move the thumb carpometacarpal joint through a range of flexion/abduction and extension/adduction**. Panels demonstrate the position of the arm **(A)** and hand **(B)**.

The CPM intervention was applied to each participant for 30 min per day over three consecutive days using a custom made device (see Figure 1). Participants sat with their right shoulder in slight abduction (10°), elbow flexed to 90° and forearm restrained in a supinated position. The thumb was supported with a flexible sling around the proximal phalanx. An AC servomotor (model YM2760, Jaycar Electronics, Adelaide, Australia) located above the device enabled variable amplitude and frequency movements to be generated in the thumb support, moving the thumb in an arc of flexion/abduction and extension/adduction. The speed (0–240° s^−1^) and amplitude (0–60°) of the movement was varied in a random fashion to prevent habituation to the passive movement with repetitive, identical stimuli. The investigator was able to alter the pattern of the passive motion from random to regular, and participants were asked to pay attention to their hand and to alert the investigator when the movement changed from random to regular. The investigator covertly altered these settings once every approximately 5 min and recorded the time interval between changing the setting and the participant reporting the change (called “detection” time). At the end of the CPM session, participants voluntarily removed their thumb from the device and replaced it on a supportive pillow for the remainder of the TMS mapping.

### Statistical Analysis

Data are presented as means ± standard deviations (SD). All parameters were analyzed for normality using Kolmogorov-Smirnov tests and log transformation was applied to the map volume data. The acute effect of CPM on TMS parameters were analyzed with Student’s paired *t*-tests (pre- vs. post-CPM on day 1). Any long-lasting effect of CPM on TMS parameters and attention were analyzed with one-way repeated measures analysis of variance (ANOVA) (time points: post CPM day 1, post-CPM day 2, post-CPM day 3, and day 8) or the non-parametric Friedman’s test for the percent change from baseline. The effect of the initial area of the motor map and attention were considered as covariates in an ANCOVA to investigate whether they influenced MEP amplitude, COG or map area. All statistical tests were performed with IBM SPSS Statistics Version 21 and the level of significance was set at *P* < 0.05.

## Results

The average age of participants was 24.3 ± 4.3 years and the average score on the Edinburgh Handedness Inventory was 87 ± 15. Group data for TMS parameters obtained before and/or after CPM are shown in Table 1. There was no difference in RMT between baseline and the end of the first CPM session (paired *t*-test, *P* = 0.47) or between days 1, 2, 3 and 8 (ANOVA, *F*_4,12_ = 0.43, *P* = 0.78).

**Table 1:** **Group data for TMS parameters and the attention task**.

	Day 1 Pre (Baseline)	Day 1 Post	Day 2 Post	Day 3 Post	Day 8
**RMT SI (%MSO)**	40.1 ± 6.7	40.4 ± 6.0	39.8 ± 6.3	39.8 ± 6.4	40.2 ± 6.7
**MEP amplitude at hot spot (mV)**	1.2 ± 0.8	0.9 ± 0.5	0.9 ± 0.6	1.0 ± 0.5	0.9 ± 0.7
**Map area (sites)**	19.1 ± 9.3	19.5 ± 9.1	18.8 ± 9.6	16.9 ± 7.6	19.9 ± 9.5
**Map volume (mV)**	4.8 ± 2.9	6.0 ± 6.4	5.1 ± 4.2	5.2 ± 5.0	6.2 ± 6.0
**Detection task time (s)**	–	59 ± 23	65 ± 24	75 ± 50	–

*RMT = resting motor threshold, SI = stimulus intensity, MSO = maximal stimulus output, MEP = motor evoked potential. The detection task was only performed during the three CPM sessions on days 1–3*.

### MEP Amplitude at the Hotspot

There was no difference between the MEP amplitude recorded at an intensity of 120% RMT at the hotspot before and after the first session of CPM on day 1 (see Table 1, paired *t*-test, *P* = 0.13). Raw (ANOVA, *F*_4,10_ = 0.67, *P* = 0.62) and normalized (percent change from baseline, Friedman’s test, Chi-square = 1.338, *p* = 0.720) MEP amplitude also did not differ across testing sessions.

### Map Area and Volume

The area of the cortical representation of APB did not differ between pre and post CPM on day 1 (see Table 1, paired *t*-test, *P* = 0.77) or across testing sessions (raw data: ANOVA, *F*_4,9_ = 0.76, *P* = 0.58; normalized data (percent change from baseline): Friedman’s test, Chi-square = 2.504, *p* = 0.475).

The APB map volume varied greatly between participants (1.1–24.5 mV) but did not significantly differ between pre- (4.8 ± 3.0 mV) and post-CPM (6.0 ± 6.4 mV) on day 1 or across testing sessions (raw data: ANOVA, *F*_4,9_ = 1.16, *P* = 0.34; normalized data (percent change from baseline): Friedman’s test, Chi-square = 3.092, *p* = 0.378).

### Center of Gravity

The COG of motor maps changed between sessions for all individuals in an inconsistent manner. The distance (mm) from the COG at baseline on day 1 was calculated, and there were no significant differences in the distance moved after the first CPM session (average COG distance moved 1.49 ± 1.42 mm) or across the remainder of the testing days (ANOVA, *F*_3,36_ = 1.047, *p* = 0.359).

### Attention

During the CPM intervention on days 1–3, the average time taken to detect a change in the pattern of CPM (i.e., index of attention) was 57.8 ± 37.6 s. Detection time did not significantly change across the six epochs within a session (ANOVA, *F*_5,14_ = 1.7, *P* = 0.16) or across testing sessions (ANOVA, *F*_2,8_ = 0.02, *P* = 0.98) and no epoch * day interaction was observed (ANOVA, *F*_10,54_ = 0.93, *P* = 0.51).

### Influence of Confounding Variables

In order to determine whether baseline map characteristics or attention influenced the response to CPM, a further analysis of variance was conducted with these variables as covariates. The initial area of the motor map had no influence on the map area (ANCOVA, *F*_4,44_ = 0.40, *P* = 0.81) or volume after CPM (ANCOVA, *F*_4,9_ = 0.63, *P* = 0.65). Likewise, attention had no influence on the post-CPM map area (ANCOVA, *F*_4,44_ = 1.88, *P* = 0.20) or map volume (ANCOVA, *F*_4,44_ = 0.48, *P* = 0.75).

## Discussion

The results of the current study suggest that single and repeated sessions of CPM of the thumb are insufficient to induce a lasting change in the representation of the APB muscle in the motor cortex of healthy adults. The lack of a change in the APB representation was evidenced by an unaltered motor map after a single session of CPM or daily sessions repeated over three consecutive days. The size of the response evoked by a specified intensity of stimulation over the APB hotspot was also unaltered suggesting unchanged excitability in this part of the motor cortex and in the APB corticomotoneuronal projection.

The lack of an acute change in the APB motor map and excitability following a single session of thumb CPM is surprising for three reasons. First, Lewis and Byblow (2004) observed a significant increase in the volume of the motor map of another muscle, the flexor carpi radialis, after a single 30-min session of passive wrist movement in healthy adults. Second, fMRI studies show that passive movement of the wrist activates primary motor cortex, again in healthy adults (Lotze et al., 2003). Lastly, two hours of tactile stimulation increases the size of the hand representation in sensory cortex in animals and improves tactile discrimination in healthy adults (Godde et al., 1996). Our results suggest that the after-effects of passive movement on the motor cortex may differ between muscles and that the mechanisms that underlie plasticity may differ for tactile stimulation and passive movement.

Our study is the first to investigate the effect of repeated sessions of passive thumb movement on the representation and excitability of thumb muscles involved in the task. However, we observed no effect of repeated sessions of thumb CPM on the representation and excitability of APB. We speculate that passive movement-induced reorganization may be better suited to muscles that have a smaller representation in the human motor cortex, like the flexor carpi radialis, or to movements that involve many muscles, like flexion and extension of the wrist. This could be investigated by comparison of modulation of APB corticomotor excitability during passive thumb movement with modulation of FCR corticomotor excitability during passive wrist flexion and extension. Alternatively, a greater number of sessions may be required to effect a change in the motor map. The observation of increased activity in motor cortices of healthy adults following 20 min of wrist movement repeated daily for 1 month (Carel et al., 2000) and enlargement of the cortical motor map following 1 h of transcutaneous electrical stimulation of the thumb daily, for 3 weeks in healthy adults (Meesen et al., 2011) suggests that this may be the case.

Factors that are known to affect experimental and use-dependent plasticity within motor cortex include attention (Stefan et al., 2004), circadian rhythm, sex, central nervous system active drugs and physical activity (see Ridding and Ziemann, 2010 for review). The current experimental design attempted to minimize any between-subject differences in attention by including an attention task and quantifying attention within- and across-sessions. No significant changes in attention were observed within- or across-sessions and lack of attention to the hand is unlikely to explain the absence of an effect on motor maps in the current study. The effect of circadian rhythm and cortisol levels (Sale et al., 2008) were minimized by scheduling experiments at the same time of day.

There are several limitations to our study. First, the average age of our participants (24.3 ± 4.3 years) was considerably younger than most people following stroke. We recruited healthy young participants because we anticipated that any effect of a motor task on cortical reorganization was most likely to be seen in younger compared with older adults (Rogasch et al., 2009). The lack of an effect in this younger sample suggests that an effect is unlikely to be present in a sample of older adults. Second, the study had a small sample size. However, the absence of even a trend towards an increase in map size is unlikely to translate into a significant effect in a larger group. Third, tactile input arising from contact between the sling and the skin on the thumb during CPM is unlike the tactile input that would normally be associated with voluntary thumb movement during for example, manipulation of objects. Finally, we did not control for or assess thumb use before the study and thus are unable to comment on the role of activities such as playing a musical instrument or prolonged video gaming on the results of the current study.

Identifying a robust and reproducible method for experimentally inducing reorganization of the sensorimotor cortex could benefit a wide range of patients. Reorganization of the sensorimotor cortex is already known to occur in natural situations such as reading Braille (Elbert and Rockstroh, 2004), learning to play a musical instrument (Elbert et al., 1995) and in response to pathological situations such as amputation (Ridding and Rothwell, 1995). A common feature is that use of a body part is associated with increased representation, while non-use, or experimentally depriving the cortex of afferent information, is associated with a decrease in the representation. It appears that change in sensory (afferent) input is a critical component of this so-called “use-dependent” plasticity (Bütefisch, 2004). Recently, some novel rehabilitation paradigms, which modify afferent input, have been developed to manipulate use-dependent plasticity in a functionally beneficial way following stroke (McDonnell et al., 2007; Schabrun and Hillier, 2009). We proposed that CPM could provide an avenue for tactile and proprioceptive input, which may help to reorganize the sensorimotor cortex and ultimately improve recovery of the hemiplegic arm post-stroke. The lack of an effect following CPM in our healthy sample may indicate that either passive movements are not functionally-meaningful enough to induce reorganization, as has been shown with some motor tasks in healthy adults (Ngomo et al., 2012), or that it is difficult to show a change in an intact, healthy motor representation. While we expect that the shortening and lengthening of the APB would have activated muscle spindles and cutaneous mechanoreceptors, the passive movement did not take the carpometacarpal joint of the thumb to the end of range and thus did not activate joint receptors. This may have contributed to the inability of the CPM to induce a lasting change.

## Conclusion

Single and repeated sessions of thumb CPM do not alter the motor cortical representation of the APB in healthy adults. Thus, use of CPM on the weak hand following stroke may not reverse the reduction in the cortical representation of the weak hand muscles or aid rehabilitation.

## Conflict of Interest Statement

The authors declare that the research was conducted in the absence of any commercial or financial relationships that could be construed as a potential conflict of interest.
